# Endovascular revascularization of chronic total arterial occlusion of the lower limb using the SAFARI technique

**DOI:** 10.1590/1677-5449.200126

**Published:** 2021-03-12

**Authors:** Miguel Alonso-Rojas Huillca, Milagros Moreno-Loaiza, Félix Tipacti-Rodríguez, Manolo Briceño-Alvarado, Wildor Samir Cubas Llalle

**Affiliations:** 1 Hospital Nacional Edgardo Rebagliati Martins – HNERM, Departamento de Cirugía de Tórax y Cardiovascular, Vascular and Endovascular Surgery Service, Lima, Peru.

**Keywords:** critical limb ischemia, subintimal angioplasty, retrograde access, popliteal artery, anterior tibial artery, isquemia crítica de membros, angioplastia subintimal, acesso retrógrado, artéria poplítea, artéria tibial anterior

## Abstract

The SAFARI technique or Subintimal Arterial Flossing with Antegrade-Retrograde Intervention is an endovascular procedure that allows recanalization of Chronic Total Occlusive (CTO) lesions when conventional subintimal angioplasty is unsuccessful. Retrograde access is usually obtained through the popliteal, anterior tibial, dorsalis pedis artery, or posterior tibial arteries and may potentially provide more options for endovascular interventions in limb salvage. The case of an 81-year-old man with a history of uncontrolled hypertension, diabetes mellitus, and dyslipidemia is presented. He presented with a cutaneous ulcer on the right lower limb with torpid evolution and poor healing. The Doppler ultrasound and arteriographic study revealed a CTO lesion of the popliteal artery that was not a candidate for antegrade endovascular revascularization, but was successfully treated using the SAFARI technique. The patient had no perioperative complications, the wound showed better healing, and he was discharged with an indication of daily dressings and control by an external outpatient clinic.

## Resumo

A técnica SAFARI, ou Subintimal Arterial Flossing with Antegrade-Retrograde Intervention, é um procedimento endovascular que permite a recanalização de lesões por oclusão total crônica (OTC) em caso de fracasso da angioplastia subintimal convencional. O acesso retrógrado é geralmente obtido através da artéria poplítea, tibial anterior, pediosa ou tibial posterior e pode fornecer mais alternativas de intervenções endovasculares para o salvamento do membro. É apresentado o caso de um homem de 81 anos com histórico de hipertenção não controlada, diabetes melito e dislipidemia. Ele apresentava uma lesão ulcerativa cutânea no membro inferior direito com evolução tórpida e má cicatrização. O ultrassom Doppler e o estudo arteriográfico revelaram uma lesão por OTC na artéria poplítea. O paciente não era candidato a revascularização endovascular anterógrada; sendo assim, esta foi realizada com successo utilizando a técnica SAFARI. O paciente não apresentou complicações perioperatórias e recebeu alta com indicação de cuidados diários com a ferida e controle em um ambulatório externo. Além disso, a ferida apresentou melhor cicatrização.

## INTRODUCTION

Due to population aging and the role of atherosclerosis, the global prevalence of Peripheral Arterial Disease (PAD) has been increasing exponentially, with an annual estimate that exceeds 200 million people affected worldwide.^1^ PAD has been classically described with long and complex lesions in the Lower Limbs (LL). However, the diffuse, multisegmental, and Chronic Total Occlusive (CTO) pattern at the infrapopliteal level in the context of Diabetes Mellitus (DM) poses multiple and significant technical challenges for the vascular surgeon. Endovascular revascularization techniques by femoral approach, antegrade, or cross-over from the contralateral limb, constitute the usual strategy for angioplasty in LL, but in about 10-20% of cases these techniques fail to recanalize CTO lesions due to inability to cross them intraluminally or to re-enter after subintimal crossing, causing imminent technical failure of these conventional techniques.^2^

It then becomes necessary to use alternative and combined techniques that involve an antegrade-retrograde approach such as Subintimal Arterial Flossing with Antegrade Retrograde Intervention (SAFARI),^3^ which together with specialized devices for crossing and reentry of CTO lesions, achieve technical success rates of 90-95%.^4^

## PART I – CLINICAL SITUATION

The patient was an 81-year-old man with a history of uncontrolled hypertension, diabetes mellitus, and dyslipidemia. He had presented with a cutaneous ulcer (4 x 6 cm) in the pretibial region of the right lower limb 8 weeks previously, with torpid evolution, poor healing, and evident signs of infection despite antibiotic treatment. Physical examination revealed that the femoral pulse was present and the popliteal, posterior tibial, and pedal pulses were absent.

**Figure 1:**
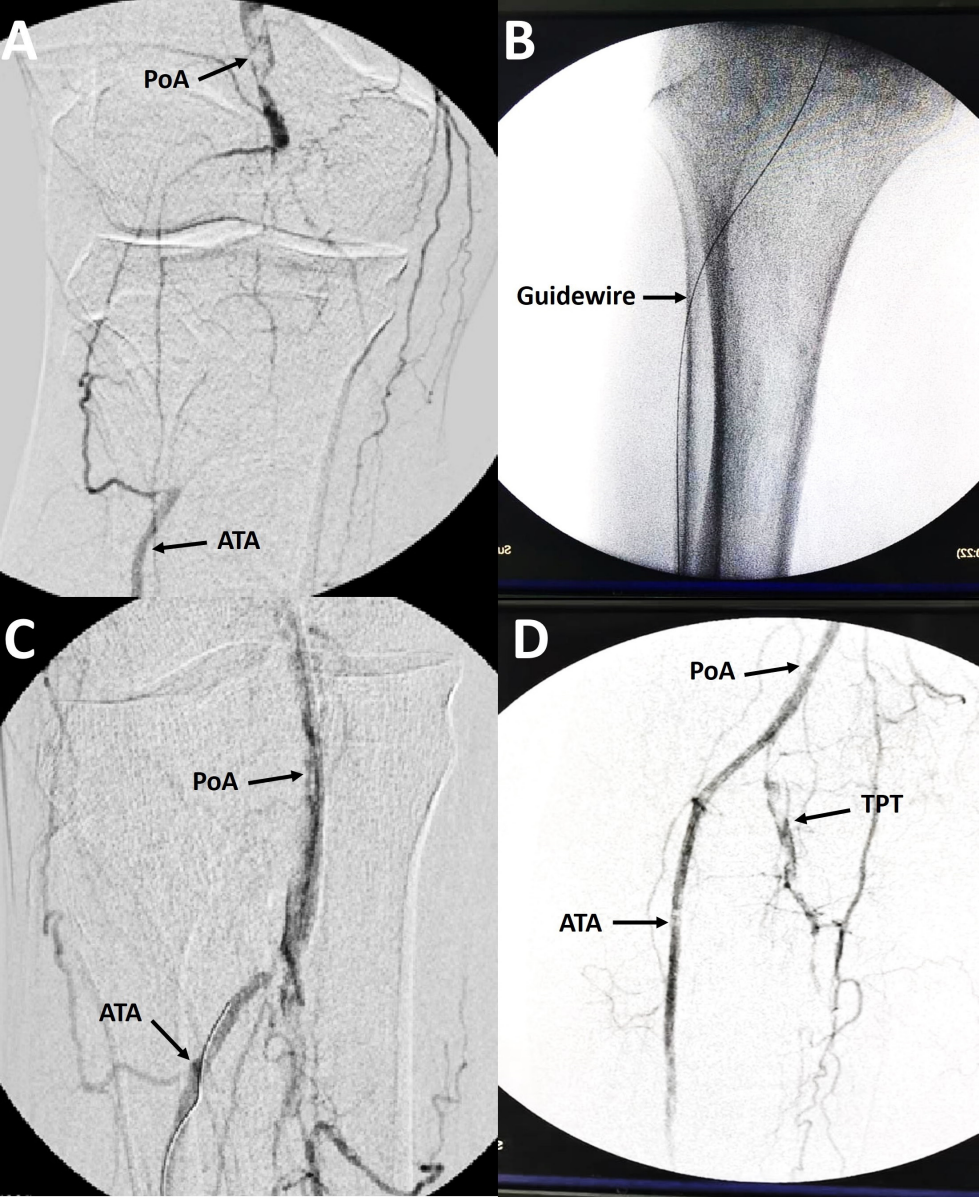
(A) CTO lesion at APo (P2) level with ATA recanalized by collaterals; (B) Crossing guidewire (0.018 “x 300 cm) advanced by retrograde approach; (C) Recanalization of the CTO (P2) lesion via the subintimal space; (D) Arteriographic control after angioplasty.

The Doppler ultrasound study revealed a biphasic wave pattern at the level of the Superficial Femoral Artery (SFA), total obstruction of the Popliteal Artery (PoA), monophasic wave in the Anterior Tibial Artery (ATA) and Dorsalis Pedis Artery (DPA), and absence of flow in the Peroneal Artery (PerA) and Posterior Tibial Artery (PTA). The arteriographic study was performed using a Common Femoral Artery (CFA) approach ipsilateral to the lesion, using an introducer and a 5 Fr Multipurpose Catheter (MPP) inserted into the distal third of the SFA. During infusion of iodinated contrast solution, a CTO lesion was identified in the P2 segment of the PoA, there was absence of flow in the Tibioperoneal Trunk (TPT), and it was observed that the ATA was recanalized by collaterals from the SFA (Figure 1A).

## PART II – WHAT WAS DONE

Given the unsuccessful attempt to traverse the CTO lesion in the PoA in an antegrade direction, a combined approach (SAFARI technique) was chosen from the distal portion of the ATA under ultrasound guidance and using a micro-puncture set and introducer 4 Fr (Figures 11C). A crossing guidewire (0.018” x 300 cm) and a support catheter (0.018”) were used for retrograde recanalization of the CTO lesion by subintimal access, returning to the true lumen at the PoA level. After passing through the occlusive lesion, the distal end of the guidewire was retrieved with the help of the MPP 5 Fr catheter previously placed in the CFA via the anterograde approach. Once the 0.018” guidewire had been successfully advanced inside the catheter in the anterograde position inserted in the subintimal space, pre-dilatation was performed with a balloon catheter (3.0 mm x 120 mm) over a support guidewire (0.014” x 300 cm), with previous administration of a bolus of 2500 U / L heparin intra-arterially.

Subsequently, the support guidewire was exchanged for a 0.035” x 260 cm wire in order to perform the PoA angioplasty with a 5mm x 120mm balloon catheter. Post-procedural arteriographic control showed flow in the PoA and indirect signs of spasm of the ATA. Intra-arterial nitroglycerin was administered in 200 µg of bolus, showing an improvement in flow and patency of the PoA, ATA, and PeA (Figure 1D). At the end of the surgical intervention, a pulse was evident in the CFA, PoA, and PeA. The patient had no perioperative complications, the wound showed better healing, and he was discharged with an indication for daily dressings and control by an external outpatient clinic.

## DISCUSSION

In PAD complicated with Chronic Limb-Threatening Ischemia (CLTI), the goal of endovascular treatment is to avoid limb loss by achieving long-term vascular permeability (relief of symptoms, healing of lesions). However, we are facing post-angioplasty patency rates close to 55-65% at 12-24 months in CTO lesions.^5^ In response to these problematic survival statistics, the need for therapeutic alternatives that offer better survival standards led to development of alternatives such as subintimal endovascular revascularization and the SAFARI technique. This method was first described by Bolia et al. in 1987 and involves creation of a new lumen through the intimal layer of the vessel wall, by advancing a guidewire around the occlusion until re-entry into the true lumen of the healthy vessel.^6^ This procedure, in conjunction with conventional ones, has enabled the era of endovascular revascularization to face the multiple challenges posed by PAD, with special regard to infragenicular vessels, and achieve better technical success rates (90-95%; 6-12 months; 95% CI, 74-100%, *p˂0.05*) and amputation-free survival.^5^^,^ 7 ^,^ 8

Recently, a study (52 patients) described frequent use of this technique in tibial-pedal access (55.7%) with a technical success rate of 60-65% (stenting was often necessary to improve these figures). During patient follow-up, a significant improvement in Ankle-Brachial Index was reported (0.54 ± 0.25 to 0.77 ± 0.25) and rates of long-term primary patency exceeded 75%.^9^ There is a subset of patients with CTO lesions at the popliteal or tibial level who are not candidates for anterograde endovascular revascularization (15-20%), but who would benefit from the SAFARI technique, being considered the best option to establish arterial flow towards the affected limb (Target Artery Path) and successfully achieving a limb salvage.^7^

Despite all the recognized benefits, this procedure has been reported to be associated with a technical failure of 13-24%, due to failure in subintimal dissection and difficulty in re-entering the true distal lumen of the vessel, causing inadvertent lengthening of the injury and involvement of collateral vessels in areas not related to the occlusion, leading to their loss and generating a high risk of limb amputation and death in inexperienced hands (2-5%).^3^^,^ 7

Finally, we highlight the importance of optimal knowledge and surgical skills on the part of the vascular and endovascular surgeon during use of the SAFARI technique; because a series of associated complications such as vascular rupture, hematoma, dissection, and even death have been reported in very exceptional cases. Therefore, it is necessary and mandatory for the surgical team to be familiar with these risks at all times and to know their respective management.^9^

## CONCLUSION

The SAFARI technique is useful in patients with complex vascular lesions and at high risk of amputation since they are candidates for retrograde subintimal recanalization when there is difficulty in entering the true distal lumen from an antegrade approach, enabling CTO lesions to be crossed and improving patency rates in the context of limb salvage. Notwithstanding what has been described, this technique requires a sufficient learning curve to obtain optimal results and we believe that it is an excellent endovascular option in cases with long and complex vascular lesions.
